# Applied Change Management in Interventional Radiology—Implementation of Percutaneous Thermal Ablation as an Additional Therapeutic Method for Small Renal Masses

**DOI:** 10.3390/diagnostics12061301

**Published:** 2022-05-24

**Authors:** Friedrich M. Lomoschitz, Harald Stummer

**Affiliations:** 1 Department of Diagnostic and Interventional Radiology, Clinic Hietzing, Wolkersbergenstrasse 1, A-1130 Vienna, Austria; 2 Institute for Management and Economics in Health Care, UMIT—University for Health Sciences, Medical Informatics and Technology, Eduard-Wallnoefer-Zentrum 1, A-6060 Hall in Tirol, Austria; harald.stummer@umit-tirol.at

**Keywords:** change management, implementation, transformation, thermal ablation, renal cell carcinoma, localized kidney cancer, small renal mass, interventional radiology

## Abstract

Interventional radiology (IR) has the potential to offer minimally invasive therapy. With this potential, new and arising IR methods may sometimes be in competition with established therapies. To introduce new methods, transformational processes are necessary. In organizations, structured methods of change management, such as the eight-step process of Kotter—(1) Establishing a sense of urgency, (2) Creating the guiding coalition, (3) Developing a vision and strategy, (4) Communicating the change vision, (5) Empowering employees for broad-based action, (6) Generating short-term wins, (7) Consolidating gains and producing more change, and (8) Anchoring new approaches in the culture—are applied based on considerable evidence. In this article, the application of Kotter’s model in the clinical context is shown through the structured transformational process of the organizational implementation of the percutaneous thermal ablation of small renal masses. This article is intended to familiarize readers in the medical field with the methods of structured transformational processes applicable to the clinical setting.

## 1. Introduction

Business organizations are, in general, familiar with structured transformational processes. However, in medicine, change is often driven by strong leadership, professional skills, or clinical necessities. Structured change management processes are, in general, rarely applied in the clinical setting. This is also true for the field of radiology.

Specifically, interventional radiology has the potential to offer alternative and minimal invasive therapeutic options. Moreover, interventional radiology works in close cooperation with clinical specialties, usually by providing auxiliary support to the specific treatments that patients actively undergo. New and arising interventional therapeutic options are often somewhat in competition with clinically established, i.e., commonly surgical, therapy. One of these fairly new procedures based on clinical evidence is the method of percutaneous, image-guided thermal ablation (TA). This technique has the potential to cure localized neoplastic masses, such as primary or metastatic lung or liver cancer as well as localized small renal cancer, by applying cold or heat. Despite the clinical evidence and the potential for a minimally invasive approach, these new interventional therapeutic options are not used as much as they could be, for various reasons. Medical therapies undergo a continuous process of change. On one side of the spectrum, for the treatment of metastatic renal cell carcinoma, there are new immune-based therapies [1,2]. On the other side of the spectrum, interventional radiology offers a minimally invasive potential treatment with curative intention for small renal masses. Hence, structured transformational processes, in addition to clinical and therapeutic evidence, may help in the implementation of new therapeutic methods. This article is intended to familiarize clinicians and interventional radiologists with the structured methods of transformation and change management, adapted for clinical needs.

## 2. Procedural Analysis

### 2.1. Definition of Small Renal Masses (SRMs)

SRMs, as a clinical entity, are defined as enhancing tumors of the kidney, <4 cm in diameter, with image characteristics consistent with stage T1aN0M0 renal cell carcinoma (RCC) [3,4,5]. Most, but not all, SRMs are RCC. The assessment must exclude metastases, in which case the patient is considered to have metastatic RCC with a small primary tumor (T1aN0M+). Regarding thermal ablation, the margin of size is even smaller, with a recommended maximum size of 3 cm [3,6,7,8].

### 2.2. Background: Diagnosis and Treatment Options of SRMs

Incidental findings of small renal masses on imaging remain the main form of RCC diagnosis in the developed world, usually through ultrasound (US), computed tomography (CT), or magnetic resonance imaging (MRI) [9].

Surgical resection is the standard treatment for locally confined small renal tumors, but minimally invasive, image-guided, percutaneous thermal ablation techniques, usually performed under CT-guidance, are increasingly being used [10].

Interventional radiology has the potential to offer minimally invasive therapy. However, the awareness of radiation doses and risks during interventional procedures is essential today in order to apply the risk-benefit assessment and to reinforce the principles of justification and optimization in clinical practice. It is in the responsibility of each individual physician, to create a culture of respect for radiation hazards and a commitment to minimize exposure and maximize protection. Thoughtful and accurate dosimetry is critical to obtain the safest and most efficacious treatment method [11,12].

Percutaneous, CT-guided thermal ablation techniques were initially performed in patients who could not undergo surgery because of comorbid illness or renal tumor recurrence, e.g., contralateral nephrectomy [10]. However, since the techniques of the method have evolved and are ongoing with the current recommendations of international urological guidelines, there is an increase in the number of patients with small renal masses who are treated by means of image-guided percutaneous thermal ablation, which is also reflected in the literature over the recent years [13,14,15,16,17,18,19,20]. Still, randomized trials comparing surgery to minimally invasive thermal ablation techniques are missing.

### 2.3. International Urological Guidelines on the Treatment of SRMs

The minimally invasive percutaneous options for the treatment of small renal masses offered by interventional radiology are increasingly included in the guidelines of international urological societies [3,6,7,8].

TA should be considered as an alternate approach for the management of cT1a renal masses <3 cm in size. For patients who elect TA, a percutaneous technique is preferred over a surgical approach whenever minimizing morbidity is feasible [6].

According to the guidelines, both radiofrequency ablation and cryoablation are options for patients who elect TA [3,6,7].

Guidelines recommend that renal mass biopsy should be performed prior to ablation to provide pathologic diagnosis and guide subsequent surveillance [3,6,7,8].

### 2.4. Commonly Used Methods of Thermal Ablation (TA) for Therapy of SRMs

Percutaneous, image-guided techniques for the ablation of small renal tumors have increasingly been used over the past two decades [14,15,16]. TA techniques were developed in an effort to improve patient procedural tolerance and reduce the potential for complications from partial nephrectomy while still preserving function [6]. A multitude of techniques and technologies have been investigated to ablate renal tumors, although radiofrequency ablation (RFA) and cryoablation (CA) have been most widely investigated and integrated into clinical practice [6,14,15,16]. 

TA has traditionally been performed through a variety of approaches, including open, laparoscopic, and percutaneous routes. Traditional concerns in the TA literature included relatively limited follow-up, lack of pre- and post-treatment biopsies to define malignancy and efficacy, and increased local recurrence rates relative to surgical excision. However, recent research conducted using a prospective study design and a long period of surveillance yielded a 10-year disease-specific survival equivalent to that reported after radical and partial nephrectomy and revealed even longer overall survival probability at five- and ten-year follow-up compared to radical or partial nephrectomy, especially in patients at higher risk [17]. 

While the superiority of RFA or CA remains controversial, it is generally accepted that the oncologic outcomes are similar for both approaches [21,22]. According to recent publications, TA by means of microwave ablation (MWA) is increasingly being used [18,19].

RFA and MWA cause localized tumor necrosis by inducing heat, at least 60–90 °C or higher, through the application of high-frequency current. CA depends on freezing the tumor tissue, lowering the temperature of the tissue to −40 °C or lower by means of a noble gas (either helium or argon). Both principles, heat and cold, lead to irreversible cell denaturation and the destruction of tumor tissue. In both techniques, an additional safety margin, usually at least 5 mm, has to be calculated to ensure complete tumor ablation.

### 2.5. Principles of Change Management and the Eight-Stage-Process of Kotter

Change occurs in today’s businesses and in healthcare on a regular basis. New technologies, new procedures, and new structural requirements are implemented regularly; some scholars and practitioners even say that ‘(strategic) management is about change and how to deal with it’. However, how to deal with change is often not thought about in medicine. Small changes often disturb routines; large changes often lead to a feeling of uncertainty within the staff, or even open or disguised resistance, especially from those involved in the change. What is change? In principle, change can be two extremes, or a mixture of the two, either: (a) ‘dropping a bombshell’, i.e., an authoritarian and secret agenda that is put into place by a hierarchy, or (b) a structured process involving the most important stakeholders creating new structures, routines, processes, and technologies at work. The latter approach is to be favored except for during crisis management and some other radical steps because it minimizes fluctuations in personnel, employee dissatisfaction, and even reactions by those employees under the influence of not-invented-here (NIH) syndrome [23].

Nearly all structured change approaches and theories use Kurt Lewin’s field theory comprising three stages of change: (1) unfreeze, (2) change, and (3) refreeze [24]; this is one method for managing change within an organization. The method involves preparing employees for change, enacting the changes, and finally, integrating and normalizing those changes within the organization. In the first phase, routines and processes have to be ‘unfrozen’; denial and resistance from employees can be expected and should be anticipated. In a participatory process, they should be involved and convinced. In the ‘moving’ phase, the processes and routines should be adopted, if possible, using a pilot study, and short-term wins for the employees should be planned. In the ‘refreezing’ phase, those processes should be re-institutionalized, whether through feedback, SOPs, or other structural leadership instruments.

**Figure 1:**
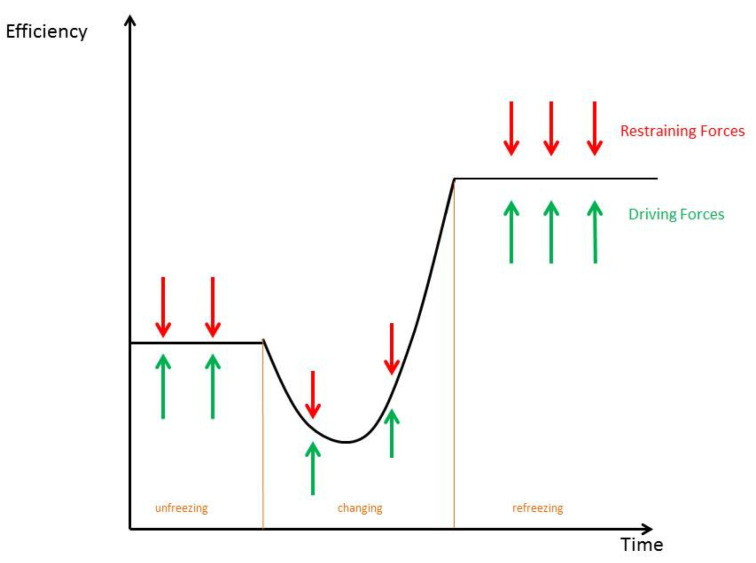
Lewin’s field model (own diagram following [24]).

As can be seen in Figure 1, in change processes, after unfreezing, efficiency normally decreases as the routines and the processes are no longer the same as what people are used to. This is the crucial part, where people need to stay convinced and to learn from the abovementioned pilot study before the changes are rolled out permanently. This traditional theory still is used nowadays, albeit in a slightly expanded form.

Actually, in research and in practice, Kotter’s adoption of Lewin’s theory is state of the art [25]. He expanded the three steps into eight [25,26,27] (Table 1).

How these steps can be used in medicine as the basis for a change in interventional radiology through the application of Kotter’s eight-step process to a model for the treatment of small renal masses with percutaneous thermal ablation techniques is discussed in the upcoming section.

## 3. Discussion

### 3.1. Establishing a Sense of Urgency

Establishing a sense of urgency is crucial to gaining the necessary cooperation [27]. Hence, a sense of urgency is the basis for every change process. In medicine in general, this urgency is immanent due to patients suffering from diseases which are potentially treatable in a curative manner, or at least through palliative care. Specifically, to introduce new treatment options with curative intentions, such as the percutaneous thermal ablation of small renal masses, colleagues in the involved fields must be aware of and familiar with the options for treatment and their associated outcomes. Scientific evidence should be the basis for new methods, and, of course, it is helpful if the recommendations are anchored in specific guidelines.

Moreover, according to Kotter, there are also different ways to raise the urgency level methodically: this ranges from using various means to “force more relevant data and honest discussion into meetings, bombarding people with information on future opportunities and on the organization’s current inability to pursue those opportunities to even creating a crisis by allowing errors to blow up instead of being corrected” [27]. Those cited management methods may be only of theoretical use in the field of medicine. However, it is of utmost importance for the initiation of a change process to share the capabilities of interventional radiology and the potential for new and minimal invasive methods with colleagues from other specialties. In the case of the TA of SRMs, this includes cooperating with urology and oncology, as well as through the collegial leadership of the hospital or medical unit and outpatient colleagues, and also through informing the public by various means. Thus, interventional radiologists have to share their specific knowledge and experience on different levels.

In the sense of the method of change management, interventional radiology can help others to see the need for change through engaged statements that communicate the importance of acting instantaneously.

### 3.2. Creating the Guiding Coalition

The first step in putting together the kind of team that can direct a change effort is to find the right membership. According to Kotter, four key characteristics seem to be essential to effectively guiding coalitions: (1) position power, (2) expertise, (3) credibility, and (4) leadership [27].

Since patients with SRMs are usually treated by urologists and TA is performed by interventional radiologists, it is necessary that members of those specialties form the main stem of the guiding coalition. Position power implies that enough key players are on board so that progress cannot easily be blocked. Collegial leadership of the unit has to be involved, usually the medical directorship, administration, and cooperating professions, e.g., technicians and nursing, have to be informed and integrated. Expertise and credibility are crucial, since the people involved have to be trusted, intelligent decisions have to be made, and actions have to be taken seriously by colleagues. Moreover, adequate proven leadership is necessary to be able to drive the change process.

Regardless of the process used, two more components are necessary: (1) trust and (2) a common goal. When trust is present, teamwork can usually be created—when trust is missing, it cannot [27]. Beyond trust, the element crucial to teamwork seems to be a common goal. Only when all members of a guiding coalition want to achieve the same objective does real teamwork become feasible [27].

In the sense of the method of change management, therefore, interventional radiology has to take a leading role in the process of implementing the new minimally invasive methods, such as the thermal ablation of small renal masses, and help to develop a trusting team with the common goal of recognizing the need for change through engaged statements that communicate the possible advantages of the method in specific conditions and for specific indications.

### 3.3. Developing a Vision and Strategy

Vision refers to a picture of the future with some implicit or explicit commentary on why people should strive to create that future [27]. A effective vision serves three important purposes: (1) clarifying the general direction, (2) motivating people to take action in the right direction, and (3) coordinating the actions of the different people.

The central part of the vision in treating a defined group of patients with small renal masses by means of percutaneous TA is that the method is minimally invasive, with low rates of associated morbidity and mortality, and, if implemented correctly, a concurrent high curative potential. Which patients are suitable for the new method and under which clinical circumstances the procedure will be performed in the upcoming future must be clarified within the various disciplines involved. Thus, the treatment of SRMs by means of percutaneous TA fulfills all characteristics that a powerful vision requires: it is (1) imaginable, (2) desirable, (3) feasible, (4) focused, (5) flexible, and (6) communicable (Table 2).

Those characteristics form an effective vision, which is an especially important factor to create a consistent strategy in a large system [27]. Hence, for the process of change or, as in this case, introduction of a modern, effective, and minimally invasive therapeutic method, it is crucial to invest our hearts, heads, and time to create an effective vision and strategy for pioneering therapies.

### 3.4. Communicating the Change Vision

The real power of a vision is unleashed only when most of those involved have a common understanding of its goals and directions [27]. In the case of introducing a new or alternative therapeutic option, it might be effective to present a well-performed example case of this new procedure. This, in combination with scientific evidence based on recent literature, may reveal the potential benefits for the patients, physicians, and medical units involved.

A key element in effective communication is the use of metaphors and examples. In radiology, this is evident and comparably easy, according to the saying “a picture is worth a thousand words”. The presentation of an effectively performed case, documented radiologically before and after treatment, with a short and simple clinical story, that can be shown repeatedly and in multiple forums may be very self-evident, self-explanatory, and helpful in communicating a vision. Moreover, seeming inconsistencies that might come up during the process, such as potential drawbacks and any adverse events of the method, have to be thoroughly addressed, discussed, and explained. In the meaning of two-way communication and adapting processes according to feedback, the potential applications of and indications for the new method can be modified to specific institutional needs and demands [27].

### 3.5. Empowering Employees for Broad-Based Action

Major transformation rarely happens unless many people assist [27]. The purpose at this stage, according to change management theory, is to remove as many barriers to the implementation of the change vision as possible so that a broad base of people is enabled to take action.

There are four constant obstacles that may hinder change processes: (1) formal structures that render action difficult, (2) a lack of required skills, (3) the personnel and information systems that render action difficult, and (4) supervisors who discourage actions aimed at the implementation of the new method.

Since this generally sounds kind of revolutionary, how can this step be adapted for the implementation of new and intended curative treatment option, such as the TA of SRMs in a specific group of patients? The five essential steps are as follows: (1) Communicate a sensible vision to the involved staff. If multidisciplinary and multiprofessional colleagues have a shared sense of purpose, it will be easier to initiate actions to implement a new and potentially beneficial method. (2) Structures have to be made compatible with the vision. Unaligned structures block necessary action. (3) Provide staff training that is required. Without the specific skills needed for the new method, colleagues feel disempowered. (4) Align the information and personnel systems to the vision. Unaligned multidisciplinary and multiprofessional systems also block action. (5) Lastly, confront supervisors who undercut required change. Concerning renal ablation, this can be based on the increasing evidence of this new method in the context of the continuously evolving change in medicine over the centuries.

### 3.6. Generating Short-Term Wins

Major change takes time—sometimes lots of time [27]. Therefore, presentation of the short-term results of a newly introduced method may provide convincing evidence that this new method works not only in theory or according to the published data but also in the setting of one’s own unit.

The presentation can be introduced at various times after the kick-off, as the presentation of a single case, of a series of the first patients, and, even better, by integrating follow-up examinations into the presentations. Those presentations can also be used to show one’s own experiences, possible risks, drawbacks, and adverse events in the individual setting, as well as to show potential modifications. The presentation of so-called ‘short-term wins’ and transparent communication strengthen the process by providing evidence that, after a lot of hard work, the sacrifices are worth it. The morale and motivation of the team can be built by positive feedback and can help to fine-tune vision and strategies as well as to undermine the blocking of potential change. Moreover, this process offers the potential that those who are more highly placed in the hierarchy are informed that the transformation is on track, and, overall, that positive momentum is being built throughout the whole institution [23].

### 3.7. Consolidating Gains and Producing More Change

Until the changed practices attain a new equilibrium and have been driven into the culture, they can be very fragile [27]. This is especially true for the phase after gaining the first promising results, even if the early stages of the transformation were successful. Whenever one lets up before the job is done, critical momentum can be lost, and regression may follow [27]. Once this possible regression begins, rebuilding momentum can be a challenging task.

Therefore, as part of the guiding coalition of the process, the credibility afforded by short-term wins has to be used to advantage. Additional people must be brought in, on multiple levels, and instructed to help with the ongoing project. The clarity of the purpose must be maintained and the process must be structured in a way that colleagues at the multidisciplinary and multiprofessional levels take responsibility for and manage specific parts of the process. At the same time, unnecessary interdependencies must be reduced and eliminated to make change easier [27]. Once more, also in this phase, sufficient leadership is crucial to the implementation of modern and promising methods such as the TA of SRMs.

### 3.8. Anchoring New Approaches in the Culture

If one thinks of antibiotic therapy or the diverse methods of minimally invasive surgery, one principle is immanent in medicine: new approaches sink into the culture only after it is very clear that they work and are superior to the old methods. This is also one of the principles of how change is anchored in a culture [27], and this principle is also generally true for the various minimally invasive methods of interventional radiology and also potentially true for the percutaneous treatment of SRMs by means of TA.

Other than dependence on the results, additional factors that anchor new processes in a culture are continuous verbal instruction and support as well as the continuous adoption of the promoting of these processes. Such actions make the new practices compatible with the culture into which they have been introduced. Finally, both changes in attitude and in behavior are very important and typically begin early in the transformational process. However, only at the end of the change cycle do most of alterations in norms and shared values become anchored in the culture [27].

## 4. Conclusions

Change management that applies structured methods to transformational processes, such as Kotter’s eight-step process, is adopted based on the considerable evidence from the organizations where it has achieved success. Since Kotter’s model is based on real life experiences and very practically orientated, it has the potential also to serve as a basic tool for the structured implementation of new, minimally invasive therapies offered by interventional radiology in addition or as alternatives to established therapies. This article is intended to familiarize radiologists and clinicians with structured models of transformational processes through the example of the implementation of the CT-guided thermal ablation of small renal masses.

## Figures and Tables

**Table 1:** Kotter’s Eight-Step Process, own table following [27].

(1) Establishing a Sense of Urgency
(2) Creating the Guiding Coalition
(3) Developing a Vision and Strategy
(4) Communicating the Change Vision
(5) Empowering Employees for Broad-based Action
(6) Generating Short-term Wins
(7) Consolidating Gains and Producing More Change
(8) Anchoring New Approaches in the Culture

**Table 2:** Creating an Effective Vision, own table following [27].

• First Draft:	The process often starts with an initial statement from a single individual reflecting both his or her dreams and real marketplace needs.
• Role of the guiding coalition:	The first draft is always modeled over time by the guiding coalition or an even larger group of people.
• Importance of teamwork:	The group process never works well without a minimum of effective teamwork.
• Role of the head and the hart:	Both analytical thinking and a lot of dreaming are essential throughout the activity.
• Messiness of the process:	Vision creation is usually a process of two steps forward and one back, movement to the left and then to the right.
• Time frame:	Vision is never created in a single meeting. The activity takes months, sometimes years.
• End product:	The process results in a direction for the future that is desirable, feasible, focused, flexible, and is conveyable in 5 minutes or less.
